# Natural history of arginase 1 deficiency and the unmet needs of patients: A systematic review of case reports

**DOI:** 10.1002/jmd2.12283

**Published:** 2022-03-25

**Authors:** Aseel Bin Sawad, Arti Pothukuchy, Mark Badeaux, Victoria Hodson, Gillian Bubb, Kristina Lindsley, Jennifer Uyei, George A. Diaz

**Affiliations:** ^1^ Aeglea BioTherapeutics, Inc. Austin Texas USA; ^2^ Health Economics and Outcomes Research ‐ Evidence Synthesis IQVIA, Inc. San Francisco California USA; ^3^ Division of Medical Genetics and Genomics, Department of Genetics and Genomic Sciences Icahn School of Medicine at Mount Sinai New York New York USA

**Keywords:** ARG1‐D, Arginase 1 deficiency, hyperargininemia, newborn screening, pegzilarginase, systematic literature review, urea cycle disorder

## Abstract

**Background:**

Arginase 1 deficiency (ARG1‐D) is a rare, progressive and debilitating urea cycle disorder characterized by clinical manifestations including spasticity, seizures, developmental delay, and intellectual disability. The aim of this systematic review was to identify and summarize the natural history of ARG1‐D and the unmet needs of patients.

**Methods:**

A comprehensive search of published case reports was undertaken to identify patients with ARG1‐D regardless of interventions, comparisons, or outcomes. MEDLINE, EMBASE, Cochrane Central Register of Controlled Trials, and other evidence‐based medicine literature databases were searched on 20 April 2020. Quality was assessed using the Joanna Briggs Institute (JBI) Critical Appraisal Checklist. (PROSPERO registration: CRD42020212142.)

**Results:**

One hundred and fifty seven ARG1‐D patients were included from 111 publications (good overall quality based on JBI's Checklist); 84 (53.5%) were males. Motor deficits (including spasticity), intellectual disability, and seizures were reported in >50% of the cases. Mean age (SD) at diagnosis was 6.4 years and the laboratory findings most commonly reported to support diagnosis included elevated plasma arginine (81.5%), mutation in *ARG1* gene through genetic testing (60%), and absence/reduction of red blood cell arginase activity (51%). Reported management approaches mainly included dietary protein restriction (68%), nitrogen scavengers (45%), and essential amino acid supplements (21%). Author‐reported clinical improvement was documented for 26% of patients, 15% deteriorated, and 19% had limited or no change; notably, no indication of clinical outcome was reported for 40% cases.

**Conclusion:**

This review illustrates a significant burden of disease and highlights a considerable unmet need for clinically effective treatment options for patients with ARG1‐D.

**SYNOPSIS**

A significant burden of disease is associated with Arginase 1 deficiency and there is a considerable unmet need for clinically effective treatment options for patients.

## 1 INTRODUCTION

Arginase 1 deficiency (ARG1‐D), a distinct urea cycle disorder (UCD), is a debilitating, progressive, inherited metabolic disease characterized by persistent elevation of plasma arginine, and is associated with considerable morbidity and early mortality.^1^, ^2^, ^3^ UCDs represent a group of inborn errors of metabolism that impair detoxification of ammonia.^3^ Arginase 1 is an enzyme that facilitates the removal of nitrogen by catalyzing arginine to ornithine in the last step of the urea cycle.^4^, ^5^, ^6^ Its absence results in excessive accumulation of plasma arginine (hyperargininemia). Excess plasma ammonia (hyperammonemia) also may occur; however, it is usually less common and less severe than in the proximal UCDs.^4^, ^5^, ^7^

Classified as a rare disorder, ARG1‐D has been estimated to occur in approximately one in 950 000 births in contrast to an overall incidence of one in 35 000 for UCDs in general.^8^ Clinical manifestations of ARG1‐D are heterogeneous and include motor deficits such as spasticity and difficulty in walking, plateauing of intellectual ability, developmental delays, and seizures.^2^, ^6^, ^9^, ^10^, ^11^, ^12^ Most infants with ARG1‐D do not exhibit any clinical manifestations during the first year of life; typically, ARG1‐D becomes evident between the ages of 1 and 3 years.^4^, ^11^ Although the onset of manifestations varies, all patients progress over time and many experience loss of motor function and neurological impairment, and have a reduced quality of life (QoL).^2^, ^4^, ^13^

ARG1‐D can be misdiagnosed as other neurological diseases such as cerebral palsy or hereditary spastic paraplegia due to similarities in clinical presentation.^3^, ^14^ Delays in diagnosis of ARG1‐D are also common.^15^, ^16^ These delays are largely due to a lack of disease awareness combined with the variability in disease manifestation. A diagnosis of ARG1‐D is usually based on the clinical profile and plasma arginine levels and confirmed by genetic testing (identification of biallelic pathogenic variants in *ARG1*) or reduced arginase enzyme activity in red blood cells (RBCs).^17^ Affected infants can be identified at birth through newborn screening,^4^ however, there are limitations with these programs (e.g., testing is not offered in many regions, the materials and equipment may not be available, and there is a lack of consistent use of appropriate analytical cut‐off values for disease indicators).^18^, ^19^ Furthermore, false negative results can occur because plasma arginine may take time to accumulate in newborns.^20^

Current treatment recommendations target reducing plasma arginine and ammonia concentrations.^4^, ^21^, ^22^ These treatment approaches include restricting dietary protein intake and administering essential amino acid [EAA] formula, plus vitamin and mineral supplementation to ensure nutritional requirements are met, while reducing the flux of nitrogen through the urea cycle. Nitrogen scavengers (such as, sodium benzoate, sodium phenylacetate, and glycerol phenylbutyrate) are often prescribed to increase ammonia excretion by providing alternative pathways for its elimination. In rare cases, ARG1‐D is treated by liver transplantation.^12^, ^21^ Under the guidance of a metabolic dietitian, food intake must be closely managed; this is often ineffective in fully controlling plasma arginine because only approximately 20–25% of arginine is derived from diet^23^ and highly restrictive diets are often difficult to adhere to.^6^, ^17^ Moreover, the recommended standard of care for ARG1‐D is based on case reports and limited clinical studies and does not address the issue of endogenous arginine production.^12^, ^21^, ^24^, ^25^

This systematic literature review (SLR) was conducted to identify the published evidence and describe the natural history of ARG1‐D, the impact of ARG1‐D on patients and their caregivers, and to examine the effectiveness of available treatments and unmet needs of patients with ARG1‐D.

## 2 METHODS

### 2.1 Overview

The methods outlined for this systematic review follow those published in the Cochrane Handbook for Systematic Reviews of Interventions,^26^ the Preferred Reporting Items for Systematic Reviews and Meta‐Analyses (PRISMA) statement,^27^ and are in accordance with the United States (US) Food and Drug Administration's (FDA) meta‐analysis draft guidance for industry.^28^ A detailed protocol was developed prior to conducting the review and was registered prospectively in PROSPERO (registration ID CRD42020212142). The PROSPERO protocol and PRISMA checklist are available as online supplementary material.

### 2.2 Study eligibility criteria and identification

Study eligibility criteria were defined in terms of population, interventions, comparisons, outcomes, and study design (PICOS). We included case reports of patients with ARG1‐D, regardless of interventions, comparisons, or outcomes.

The search was designed to be comprehensive and capture all case reports of patients with ARG1‐D. Terms for UCDs also were added to capture studies not indexed under ARG1‐D or hyperargininemia. Multiple databases were searched to identify relevant evidence including Medical Literature Analysis and Retrieval System Online (MEDLINE), Excerpta Medica database (EMBASE), Cochrane Central Register of Controlled Trials, Cochrane Database of Systematic Reviews, the Health Technology Assessment Database, EconLit, and National Health Service (NHS) Economic Evaluation Database. The searches were executed on April 20, 2020 according to the systematic search strategies; the language of publication was restricted to English (complete search strategies and results are presented in supplementary materials). To supplement the searching of bibliographic databases, reference lists of relevant studies were reviewed to identify additional citations of case reports and clinicaltrials.gov was searched for recent and ongoing studies. An update of the search was conducted on July 6, 2021 to identify new publications of case reports and assess whether recent publications would impact the results of the SLR.

### 2.3 Study selection

All unique publications identified through the systematic review were evaluated in a two‐step process to assess whether they were eligible for data extraction based on pre‐defined inclusion and exclusion criteria. First, two reviewers independently reviewed all unique titles and abstracts identified by the search. Records assessed as not relevant by both reviewers were excluded; records assessed by both reviewers as definitely or possibly relevant were retained for full‐text review. Second, the eligibility of each full‐text publication was assessed by two independent reviewers, and reasons for exclusion were documented. At any stage, a third reviewer adjudicated a decision to include or exclude those publications for which there was uncertainty regarding their eligibility for inclusion.

### 2.4 Data collection, quality assessment, and synthesis

A data extraction template was developed and piloted to capture study characteristics, patient demographics, methods of diagnosis, clinical manifestations, treatment characteristics, and outcomes for all included studies. Data extraction was conducted by two independent reviewers and reconciled against the source document by a third reviewer in case of any discrepancy.

Data were extracted and categorized as reported by the study authors and confirmed by a clinical expert (G.A.D.). For instances where information was available, but no formal assessment was made by the study authors, a clinical expert (G.A.D.) reviewed the information and provided an assessment if appropriate. For example, if brain imaging results were not reported as normal or abnormal by the study authors, the clinical expert reviewed the results and classified the information. The assessments were reviewed and confirmed by coauthors. The SLR results that incorporate clinical expert assessments are presented in addition to the results reported within the publications.

A quality assessment of individual papers was performed using the Joanna Briggs Institute (JBI) Critical Appraisal Checklist for Case Reports (supplementary materials).^29^ For each of the eight domains in the checklist, two reviewers independently determined whether the domain was clearly discussed or described in the study report. For any domain for which there was a discrepancy between reviewers, a third reviewer adjudicated the final assessment.

Data were synthesized descriptively as proportions, means, and medians as appropriate.

## 3 RESULTS

### 3.1 SLR summary and PRISMA

**FIGURE 1:**
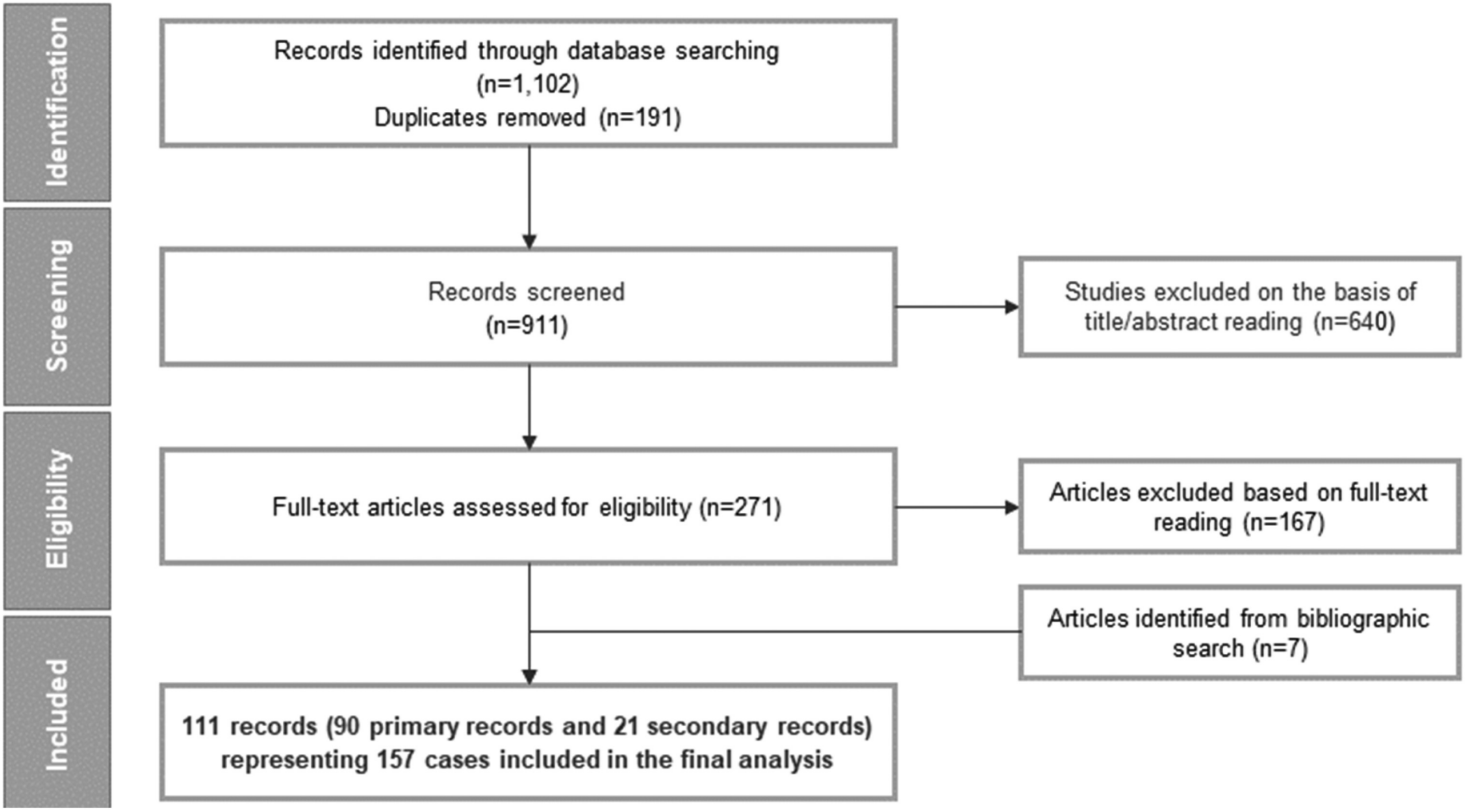
PRISMA flow diagram

The database searches identified a total of 1102 records. Following deduplication, 911 records underwent title and abstract screening, of which 271 records were retained for full‐text review. A total of 104 records meeting the eligibility criteria and seven additional records identified from bibliography searches were included after full‐text review. Overall, 111 publications (90 primary and 21 secondary records) reporting data on 157 unique cases with ARG1‐D were included in the review (Figure 1).

### 3.2 Study characteristics and patient demographics

Out of 111 included publications, 39 (35.1%) were from Asia; 35 (31.5%) from Europe; 32 (28.8%) from North America; four (3.6%) from South America; and one publication from Australia (Table S1). Of the 157 included patients, 84 (53.5%) were males and 51 (32.4%) were born of known consanguineous parentage. Ethnicity/race was reported for 80 patients, of which 31 (19.7%) were Latino, 19 (12.1%) Asian, 11 (7.0%) Middle Eastern, 10 (6.3%) European/White descent, 6 (3.8%) Malay, and 3 (1.9%) African descent/Black (Table 1).

**TABLE 1:** Patient demographics

Patient demographics	
Gender	
Male, *n* (%)	84 (53.5)
Female, *n* (%)	68 (43.3)
Not reported, *n* (%)	5 (3.1)
Mean age at diagnosis (*n* = 99), years (standard deviation)	6.4 (5.8)
Parental consanguinity	
Yes, *n* (%)	51 (32.4)
No, *n* (%)	58 (36.9)
Not reported, *n* (%)	48 (30.5)
Ethnicity/race	
African descent/Black, *n* (%)	3 (1.9)
Asian, *n* (%)	19 (12.1)
European/White, *n* (%)	10 (6.3)
Hispanic/Latino, *n* (%)	31 (19.7)
Middle Eastern, *n* (%)	11 (7.0)
Malay, *n* (%)	6 (3.8)
Not reported/not specified, *n* (%)	77 (49.0)

### 3.3 Clinical manifestations and natural history

**FIGURE 2:**
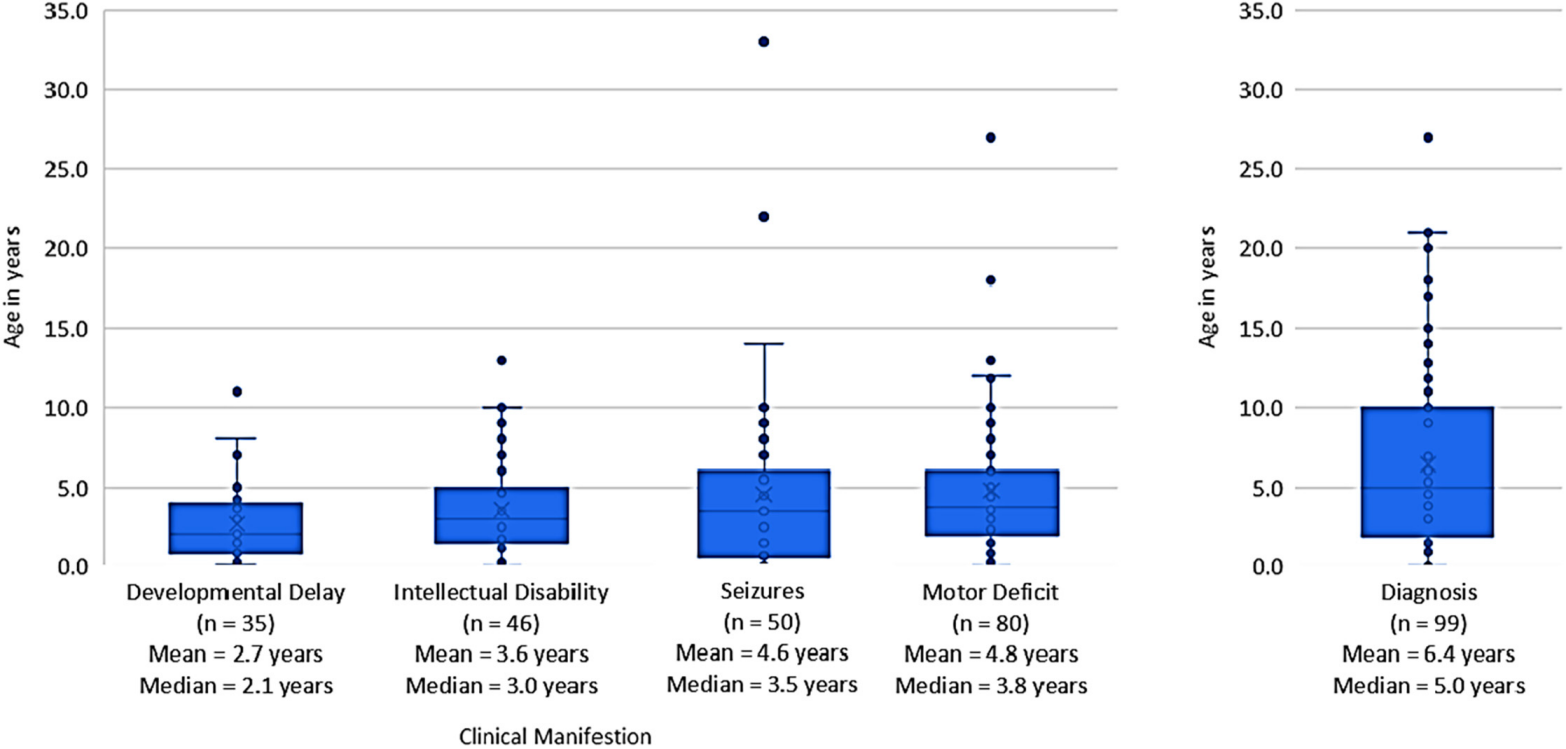
Age of onset of clinical manifestations and diagnosis

General motor deficits (including spasticity; impaired mobility/gait; impaired balance/ataxia), intellectual disability, and presence of seizures were the most commonly reported clinical manifestations of ARG1‐D (present in >50% cases). Other clinical manifestations of ARG1‐D included developmental delay (*n* = 58, 36.9%), impaired mobility/gait (*n* = 60, 38.2%), vomiting (*n* = 47, 29.9%), microcephaly (*n* = 24, 15.2%), and somatic growth delay (*n* = 35, 22.2%) (Table 2). Clinical manifestations most commonly appeared by the age of 0–3 years among cases reporting age of onset; however, it is worth noting that age of onset was not reported for 37% of the cases. Developmental delays were the earliest observed manifestations (mean 2.7 years; median 2.1 years), followed by intellectual disabilities (mean 3.6 years; median 3.0 years), seizures (mean 4.6 years; median 3.5 years), and motor deficits (mean 4.8 years; median 3.8 years) (Figure 2).

**TABLE 2:** Clinical manifestations of 157 included patients

Clinical manifestations	Author‐reported assessment				Clinical expert assessment^a^			
	Yes	No	Some information	Not reported	Yes	No	Some information	Not reported
Developmental delay, *n* (%)	58 (36.9)	13 (8.3)	17 (10.8)	69 (43.9)	65 (41.4)	16 (10.2)	7 (4.5)	69 (43.9)
Onset of developmental delay by age ≤3 years, *n* (%)	24 (41.4)	–	–	23 (39.7)	25 (38.5)	–	–	29 (18.5)
Onset of developmental delay by age >3 and ≤5 years, *n* (%)	8 (13.8)	–	–		8 (5.1)	–	–	
Onset of developmental delay by age >5 years, *n* (%)	3 (5.2)	–	–		3 (1.9)	–	–	
Intellectual disability	87 (55.4)	16 (10.2)	9 (5.7)	45 (28.7)	92 (58.6)	18 (11.5)	2 (1.3)	45 (28.7)
Onset of intellectual disability by age ≤3 years, *n* (%)	27 (31.0)	–	–	41 (47.1)	27 (29.3)	–	–	46 (50.0)
Onset of intellectual disability by age >3 and ≤5 years, *n* (%)	11 (12.6)	–	–		11 (12.0)	–	–	
Onset of intellectual disability by age >5 years, *n* (%)	8 (9.2)	–	–		8 (8.7)	–	–	
Motor deficits, *n* (%)	126 (80.3)	4 (2.5)	3 (1.9)	24 (15.3)	127 (80.9)	5 (3.2)	1 (0.6)	24 (15.3)
Onset of motor deficits by age ≤ 3 years, *n* (%)	38 (30.2)	–	–	46 (36.5)	38 (29.9)	–	–	47 (37.0)
Onset of motor deficits by age >3 and ≤5 years, *n* (%)	19 (15.1)	–	–		19 (15.0)	–	–	
Onset of motor deficits by age >5 years, *n* (%)	23 (18.3)	–	–		23 (18.1)	–	–	
Spasticity, *n* (%)	108 (68.8)	11 (7.0)	0	38 (24.2)	108 (68.8)	11 (7.0)	0	38 (24.2)
Spastic paraplegia (lower limbs), *n* (%)	102 (65.0)	11 (7.0)	1 (0.6)	43 (27.4)	102 (65.0)	11 (7.0)	1 (0.6)	43 (27.4)
Spastic quadriplegia (lower and upper limbs), *n* (%)	25 (15.9)	15 (9.6)	8 (5.1)	109 (69.4)	29 (18.5)	15 (9.6)	3 (1.9)	110 (70.1)
Progressive spasticity, *n* (%)	12 (7.6)	0	0	145 (92.4)	12 (7.6)	0	0	145 (92.4)
Impaired mobility/gait, *n* (%)	60 (38.2)	1 (0.6)	9 (5.7)	87 (55.4)	66 (42)	1 (0.6)	3 (1.9)	87 (55.4)
Seizures, *n* (%)	78 (49.7)	19 (12.1)	4 (2.5)	56 (35.7)	83 (52.9)	18 (11.5)	0	56 (35.7)
Onset of seizures by age ≤3 years, *n* (%)	24 (30.7)	–	–	28 (35.8)	26 (31.3)	–	–	31 (37.3)
Onset of seizures by age >3 and ≤5 years, *n* (%)	12 (15.3)	–	–		12 (14.4)	–	–	
Onset of seizures by age >5 years, *n* (%)	14 (17.9)	–	–		14 (16.8)	–	–	
Vomiting, *n* (%)	47 (29.9)	9 (5.7)	2 (1.3)	99 (63.1)	49 (31.2)	9 (5.7)	0	99 (63.1)
Somatic growth delay, *n* (%)	35 (22.3)	3 (1.9)	19 (12.1)	100 (63.7)	39 (24.8)	14 (8.9)	4 (2.5)	100 (63.7)
Microcephaly, *n* (%)	24 (15.3)	6 (3.8)	22 (14.0)	105 (66.9)	27 (17.2)	25 (15.9)	0	105 (66.9)

Abbreviation: NA, not applicable.

^a^ Clinical expert assessment entailed confirming the author‐reported assessment classifications and classifying data with some information as “Yes” or “No.” An example of “some information” included case reports was when head circumference measurements were reported, but not assessed as normal or abnormal by the authors. For these cases, the clinical expert assessed whether microcephaly was present or absent based on review of patient characteristics (e.g., age) and clinical description.

#### 3.3.1 Comparison with clinical expert assessments

Motor deficits (*n* = 127, 80.9%) including spasticity (*n* = 108, 68.8%), intellectual disability (*n* = 92, 58.6%), and presence of seizures (*n* = 83, 52.9%) were also the most common clinical manifestations per clinical expert assessments (Table 2). The proportions of clinical manifestations increased by up to 4% compared with original reviewer extractions since data classified as “some information” from the publication could be further classified by the clinical expert.

### 3.4 Laboratory values and measures of investigation

The most common laboratory findings evaluated in ARG1‐D patients were elevation in plasma arginine levels (*n* = 128, 81.5%) and elevated blood ammonia levels (*n* = 80, 51.0%). Genetic testing was used in 94 (59.9%) patients and measuring the absence of arginase in RBCs was used in 80 (51.0%) patients to confirm diagnosis (Table 3). The mean (standard deviation [SD]) age of diagnosis was 6.4 years (5.8) (Figure 2).

**TABLE 3:** Diagnostic and lab measures of 157 included patients

Lab findings and procedures	Author‐reported assessment				Clinical expert assessment			
	Yes, *n* (%)	No, *n* (%)	Some information, *n* (%)	Not reported, *n* (%)	Yes, *n* (%)	No, *n* (%)	Some information, *n* (%)	Not reported, *n* (%)
Elevated plasma arginine	128 (81.5)	1 (0.6)	0	28 (17.8)	NA	NA	NA	NA
Elevated blood ammonia	80 (51.0)	26 (16.6)	2 (1.3)	49 (31.2)	NA	NA	NA	NA
Absence or reduction of RBC arginase activity	80 (51.0)	0	8 (5.1)	69 (43.9)	82 (52.2)	1 (0.6)	5 (3.2)	69 (43.9)
Genetic testing	94 (59.9)	5 (3.2)	5 (3.2)	53 (33.1)	95 (60.5)	9 (5.7)	0	53 (33.8)
Abnormal EEG	43 (27.4)	6 (3.8)	12 (7.6)	96 (61.1)	52 (33.1)	9 (5.7)	0	96 (61.1)
Abnormal brain imaging	44 (28.0)	11 (7.0)	10 (6.4)	92 (58.6)	47 (29.9)	14 (8.9)	4 (2.5)	92 (58.6)
Cerebral atrophy	32 (20.4)	3 (1.9)	4 (2.5)	118 (75.2)	34 (21.7)	5 (3.2)	0	118 (75.2)

Abbreviations: EEG, electroencephalogram; NA, not applicable; RBC, red blood cell.

Other reported procedures conducted in this patient group included electroencephalogram (EEG), which was reported as abnormal for 43 patients (27.4%) and brain imaging, which was reported as showing any brain abnormality for 44 patients (28.0%), most of whom had evidence of cerebral atrophy (*n* = 32, 20.4%).

#### 3.4.1 Comparison with clinical expert assessments

After assessment by the clinical expert, the proportion of patients positive for each laboratory findings increased slightly (1–6%) compared with original author‐based data extraction: genetic testing (95 patients, 60.5%), absence of RBC arginase activity (82 patients, 52.2%), abnormal EEG (52 patients, 33.1%), abnormal brain imaging (47 patients, 29.9%), and cerebral atrophy (34 patients, 21.7%) (Table 3).

### 3.5 Treatment and management

Dietary protein restriction, either physician prescribed (as standard of care) or patient self‐selected (protein aversion), was the most frequently reported intervention (68% of patients). Nitrogen scavengers, namely sodium benzoate, sodium phenylacetate, and sodium phenylbutyrate, were administered to 70 (44.5%) patients. Less commonly reported treatments included EAA supplementation (*n* = 33, 21.0%), dialysis (*n* = 8, 5.0%), and liver transplantation (*n* = 5, 3.1%) (Table S2). Of the five patients who underwent liver transplantation, none were reported to have died and three achieved normalization of arginine levels up to 10 years after transplantation.

### 3.6 Clinical outcomes and unmet needs

Author‐reported assessments indicated global clinical outcome improvement in 41 (26.1%) patients, whereas 24 (15.3%) patients declined and 30 (19.1%) patients had no change or limited change; no author‐reported outcome assessment was provided for 62 (39.5%) patients (Table 4). Clinical outcomes specific to cognitive function (*n* = 41, 26.1%), motor function (*n* = 40, 25.4%), and somatic growth (*n* = 23, 14.6%) were reported for only a small percentage of patients. Hospitalization was reported for 48 (30.6%) patients and 16 (10.2%) were reported as deceased at the time of publication (median age 5.7 years). Of 16 patients who died, the reported causes of death included cardiac arrest, cerebral edema, pneumonia/respiratory complications, and/or sepsis. A notable proportion of outcome data (74–85%) were not reported across all clinical outcomes (Table 4). None of the included studies reported information about caregiver or QoL outcomes.

**TABLE 4:** Summary of outcomes—Functional changes after treatment

	Author‐reported assessment				Clinical expert assessment		
	Author‐reported global outcome, *n* (%)	Somatic growth, *n* (%)	Motor function, *n* (%)	Cognitive function, *n* (%)	Somatic growth, *n* (%)	Motor function, *n* (%)	Cognitive function, *n* (%)
Improved	41 (26.1)	6 (3.8)	11 (7.0)	15 (9.6)	10 (6.4)	17 (10.8)	24 (15.3)
Declined	24 (15.3)	1 (0.6)	5 (3.2)	1 (0.6)	1 (0.6)	11 (7.0)	5 (3.2)
No change/limited change	30 (19.1)	3 (1.9)	7 (4.5)	3 (1.9)	4 (2.5)	12 (7.6)	10 (6.4)
Some information	0	13 (8.3)	17 (10.8)	22 (14.0)	8 (5.1)	0	2 (1.3)
NR	62 (39.5)	134 (85.4)	117 (74.5)	116 (73.9)	134 (85.4)	117 (74.5)	116 (73.9)

Abbreviation: NR, not reported.

### 3.7 Search update

An update of the searches to capture studies published since the date of the last search (April 20, 2020) to July 06, 2021 identified 6 new relevant case studies reporting 13 individual cases with *ARG1* mutations.^30^, ^31^, ^32^, ^33^, ^34^, ^35^ The results from these studies were consistent with the main findings presented in this review and further support the burden of ARG1‐D and the need for effective treatments.

### 3.8 Quality assessment of included studies

Using the JBI Critical Appraisal Tool for Case Reports, the overall quality of 90 included primary records was generally good, with >60% records scoring “yes” for most domains (Figure S1). The adverse event domain was poorly reported across almost all records (89 of 90 records). The detailed quality assessment for each primary record can be found in the supplementary materials.

## 4 DISCUSSION

Among 157 patients identified from published case reports, the natural history of ARG1‐D was reflected in a multitude of clinical manifestations, such as developmental delay, intellectual disability, motor deficits including spasticity and impaired mobility, and seizures. Developmental delay was reported between 1 and 3 years of age, followed by intellectual disability, seizures, and motor deficits by 5 years of age. Diagnosis was often delayed compared with the onset of manifestations. Dietary protein restriction was the most frequently reported treatment. Current standard of care for ARG1‐D included severe dietary protein restriction, EAA, and ammonia scavengers. A lack of clinical improvement with standard treatments were reportedly due to difficulty with adherence as well as failure to reach plasma arginine therapeutic goals.

To our knowledge, this is the first systematic review to summarize the natural history of patients with ARG1‐D. Similar to previous research,^13^, ^17^, ^36^ this review found that the hallmark disease indicators of ARG1‐D include persistently elevated plasma arginine, the absence or reduction of arginase activity in RBCs, and a high proportion of disabling motor deficits, particularly spasticity of the lower limbs. Also consistent with previous research is that the clinical manifestations of ARG1‐D tend to be absent during newborn and infant ages, and typically first appear during ages 1–3 years.^9^, ^10^, ^13^, ^37^ The geographic and ethnic composition of patients published in literature (20% Hispanic/Latino, 12% Asia, 7% Middle East) is consistent with known genetic profiles in which the most common ARG1‐D mutations cluster in Brazil, China, and Turkey (the founder variant is from French Canada).^38^, ^39^ The disease also was shown to be progressive, with many patients declining and/or showing regression of developmental milestones. Given the progression of ARG‐1 in the first years of life and frequent delays in diagnosis, early detection provides an opportunity for earlier intervention and positive impact later in life.^38^, ^40^, ^41^

Although clinical practice guidelines are available for UCDs generally, those specific to the care and management for ARG1‐D are limited and tend to closely mirror the recommendations for other UCDs.^12^ However, focused clinical practice guidelines for ARG1‐D are important because ARG1‐D manifests differently compared with other types of UCDs, especially with respect to lower rates of hyperammonemia. Dietary protein restriction was the most commonly reported intervention among included case reports, which is consistent with medical consensus to restrict dietary protein intake for the majority of patients to reduce the arginine and ammonia levels in the plasma. In some patients, EAA may be prescribed to supplement dietary restrictions or nitrogen scavengers may be prescribed to reduce risk of hyperammonemia. In very severe cases, liver transplantation may be considered; 3% of the patients included in this review underwent liver transplantation at the time of the publication.^5^ Unfortunately, of these currently available treatment options, none directly address the underlying disease and there is a paucity of published evidence showing their clinical effectiveness over time. More recently, newborn screening for ARG1‐D has become more widespread, and it is possible that earlier detection and initiation of treatment could lead to improved clinical outcomes.^4^ However, among the 157 cases included in this review, only seven were diagnosed by newborn screening (all published in 2009 or after).

The lack of approved therapies and poor outcomes with standard of care for ARG1‐D highlight a clear unmet need for clinically effective treatment options for patients with ARG1‐D. Only 15–26% of case studies reported clinical outcomes after any treatment, with only a minority of studies reporting any improvement in clinical outcomes, highlighting that the current standard of care alone is inadequate, and the vast majority of patients fail to experience sustained clinical benefit. Among the 157 included cases, clinical manifestations such as motor deficits, intellectual disability, and seizures were common, yet few treatment options were being utilized to address the underlying disease and none of the current options address the absence or reduction of arginase activity needed to reduce the level of arginine in patients with ARG1‐D. Novel treatment options addressing the underlying disease of ARG1‐D, such as the human enzyme therapy pegzilarginase, could have the potential to improve clinical outcomes and reduce disease manifestations in patients with ARG1‐D.^42^ The FDA granted breakthrough therapy designation to pegzilarginase for the treatment of ARG1‐D in 2019^43^ based on results of a phase I/II clinical trial (NCT02488044) that suggest treatment with pegzilarginase rapidly and sustainably reduces plasma arginine levels in patients with ARG1‐D to within treatment guideline levels.^44^, ^45^, ^46^

There are some considerations that may limit the interpretation of the results from this SLR. First, data were derived from case reports only, which are considered low‐level evidence. However, it is acknowledged that case reports play an important role in elucidating information about rare disorders because conducting randomized clinical trials (RCTs) or observational studies with large sample sizes are often not feasible for rare diseases.^47^ Second, inconsistent and heterogeneous reporting of information across the studies limit the conclusions that can be reached based on this sample of cases and indicate a need for greater adherence to reporting guidelines for case studies, such as the CARE (CAse Reporting) guidelines.^48^ There were large proportions of data elements (diagnostic test, intervention, or outcomes) that were not reported by individual studies, which could lead to underestimation of their frequency. For instance, EAA was reported for 21% of patients while dietary protein restriction was reported for 68% of patients. It is well recognized that nearly all patients with ARG1‐D require management with protein restriction to minimize arginine intake and the majority of these patients receive supplementation of EAA to maintain nutritional status.^2^, ^12^, ^15^ Furthermore, the presence of some clinical manifestations may be underestimated due to differences in definitions and descriptions across case reports. For example, spasticity or impaired mobility in patients may have been reported only more generally as having motor deficits in the case reports; hence, data for these patients would have been extracted as “not reported” for the specific categories of spasticity and impaired mobility. Rare disease patient registries, such as the Network for Intoxication type Metabolic Diseases (E‐IMD), could play an important role in the collection and standardization of natural history, clinical, and therapeutic data.^49^ Finally, no date restriction was employed for including cases in this review. The included case reports span five decades (1972–2020). Changes in clinical practice and developments in the understanding of ARG1‐D over time may have an impact on the treatment and management of patients resulting in overall better outcomes of more recent cases irrespective of specific therapy. However, regardless of the date of publication, evidence of the clinical effectiveness of current treatment modalities was lacking.

## 5 CONCLUSIONS

In published case reports, the most commonly reported treatment approach for ARG1‐D was a highly restrictive diet, followed by nitrogen scavengers, and EAA supplements, which resulted in many patients showing little to no improvement after onset of disease manifestations, and many experiencing declining motor and cognitive function, hospitalization, and/or premature death. This systematic literature review corroborates findings from previous publications and highlights the high burden of disease and large unmet medical need in ARG1‐D. The development of human enzyme therapy, such as pegzilarginase, may be an important step to treating the underlying disease, lowering plasma arginine to target levels, and preventing disease progression.

## CONFLICT OF INTEREST

Aseel Bin Sawad is an employee of Aeglea BioTherapeutics.

Arti Pothukuchy is an employee of Aeglea BioTherapeutics.

Mark Badeaux is an employee of Aeglea BioTherapeutics.

Victoria Hodson is an employee of Aeglea BioTherapeutics.

Gillian Bubb is an employee of Aeglea BioTherapeutics.

Kristina Lindsley is an employee of IQVIA, which provides consulting and other research services to biopharmaceutical companies and received funding from Aeglea BioTherapeutics to conduct this study.

Jennifer Uyei is an employee of IQVIA, which provides consulting and other research services to biopharmaceutical companies and received funding from Aeglea BioTherapeutics to conduct this study.

George A. Diaz works at the Division of Medical Genetics and Genomics in the Department of Genetics and Genomic Sciences at the Icahn School of Medicine at Mount Sinai and is an external consultant with Aeglea BioTherapeutics.

## ETHICS STATEMENT

This article does not contain any studies with human or animal subjects performed by the any of the authors.

## Supporting information


**Data S1**: Supporting Information

## Data Availability

All data that support the findings of this study are present in the paper. Additional data related to this paper may be requested from the corresponding author, A.B., upon reasonable request.
