# Supplementary material for: Natural history of arginase 1 deficiency and the unmet needs of patients: A systematic review of case reports
Source: JIMD Rep. 2022 Mar 25;63(4):330–40. doi: 10.1002/jmd2.12283 (PMC9259395; doi:10.1002/jmd2.12283)
Supplement: Supplementary file 1 — Data S1: Supporting Information [file JMD2-63-330-s001.docx]

Supplement 7. Additional Tables and Figures

Natural History of Arginase 1 Deficiency and the Unmet Needs of Patients: A Systematic Review of Case Reports

**Authors:**

Aseel Bin Sawad^1^, Arti Pothukuchy^1^, Mark Badeaux^1^, Victoria Hodson^1^, Gillian Bubb^1^, Kristina Lindsley^2^, Jennifer Uyei^2^, George A. Diaz^3^

**Affiliations:**

1 Aeglea BioTherapeutics, Inc.

2 IQVIA, Inc.

3 Division of Medical Genetics and Genomics in the Department of Genetics and Genomic Sciences at the Icahn School of Medicine at Mount Sinai

**Corresponding author name and details:** Aseel Bin Sawad, Aeglea BioTherapeutics, Inc., abinsawad@aeglea.com

**Table S1. Study characteristics (country)**

| STUDY CHARACTERISTICS | | |
| --- | --- | --- |
| **Continent and corresponding publications, n (%)** | **Country** | **Case included per country, n (%)** |
| Asia, 39 (35.1) | Japan | 16 (14.4) |
|  | Turkey | 8 (7.2) |
|  | India | 5 (4.5) |
|  | China | 4 (3.6) |
|  | Bahrain | 1 (0.9) |
|  | Oman | 1 (0.9) |
|  | UAE | 1 (0.9) |
|  | Israel | 1 (0.9) |
|  | Korea | 1 (0.9) |
|  | Malaysia | 1 (0.9) |
| Australia, 1 (0.9) | Australia | 1 (0.9) |
| Europe, 35 (31.5) | Germany | 9 (8.1) |
|  | Italy | 7 (6.3) |
|  | Portugal | 7 (6.3) |
|  | France | 3 (2.7) |
|  | United Kingdom | 2 (1.8) |
|  | Spain | 2 (1.8) |
|  | Belgium | 2 (1.8) |
|  | Netherlands | 1 (0.9) |
|  | Switzerland | 1 (0.9) |
|  | Austria | 1 (0.9) |
| North America, 32 (28.8) | United States | 25 (22.5) |
|  | Canada | 6 (5.4) |
|  | Guatemala | 1 (0.9) |
| South America, 4 (3.6) | Brazil | 4 (3.6) |

**Table S2. Summary of treatments used across 157 included patients**

| Treatments | | n (%) |
| --- | --- | --- |
| Dietary protein restriction | Physician prescribed | 97 (61.7) |
|  | Self-selected | 9 (5.7) |
|  | No | 1 (0.6) |
|  | NR | 50 (31.8) |
| Nitrogen scavengers* | Yes | 70 (44.5) |
|  | No | 2 (1.2) |
|  | NR | 85 (54.1) |
| Essential amino acids | Yes | 33 (21.0) |
|  | No | 1 (0.6) |
|  | NR | 123 (78.3) |
| Dialysis | Yes | 8 (5.0) |
|  | No | 0 |
|  | NR | 149 (94.9) |
| Liver transplantation | Yes | 5 (3.1) |
|  | No | 1 (0.6) |
|  | NR | 151 (96.1) |

**Includes sodium benzoate, sodium phenylacetate and sodium phenylbutyrate; NR: Not reported*

**Figure S1. Quality of included case reports using JBI critical appraisal tool**

*JBI: Joanna Briggs Institute*
